# The imitation game: large language models versus multidisciplinary tumor boards: benchmarking AI against 21 sarcoma centers from the ring trial

**DOI:** 10.1007/s00432-025-06304-9

**Published:** 2025-09-10

**Authors:** Cheng-Peng Li, Aimé Terence Kalisa, Siyer Roohani, Kamal Hummedah, Franka Menge, Christoph Reißfelder, Markus Albertsmeier, Bernd Kasper, Jens Jakob, Cui Yang

**Affiliations:** 1 Key Laboratory of Carcinogenesis and Translational Research (Ministry of Education/Beijing), Sarcoma Center, Peking University Cancer Hospital & Institute, Beijing, China; 2 Department of Surgery, Mannheim School of Medicine, Medical Faculty Mannheim, Heidelberg University, Mannheim, Germany; 3 Department of Radiation Oncology, Charité-Universitätsmedizin Berlin, Corporate Member of Freie Universität Berlin and Humboldt-Universität Zu Berlin, Berlin, Germany; 4 BIH Charité (Junior) Clinician Scientist Program, Berlin Institute of Health at Charité-Universitätsmedizin Berlin, BIH Biomedical Innovation Academy, Berlin, Germany; 5 German Cancer Consortium (DKTK), Partner Site Berlin, a Partnership Between DKFZ and Charité-Universitätsmedizin Berlin, Berlin, Germany; 6 Mannheim School of Medicine, DKFZ-Hector Cancer Institute, Medical Faculty Mannheim, Heidelberg University, Mannheim, Germany; 7 Department of General, Visceral and Transplantation Surgery, Ludwig-Maximilians-Universität (LMU) Munich, LMU University Hospital, Munich, Germany; 8 Sarcoma Unit, Mannheim Cancer Center (MCC), Mannheim University Medical Center, University of Heidelberg, Mannheim, Germany

**Keywords:** Large language model, Clinical decision, Artificial intelligence, Soft tissue sarcoma, Multidisciplinary tumor board

## Abstract

**Purpose:**

The study aims to compare the treatment recommendations generated by four leading large language models (LLMs) with those from 21 sarcoma centers’ multidisciplinary tumor boards (MTBs) of the sarcoma ring trial in managing complex soft tissue sarcoma (STS) cases.

**Methods:**

We simulated STS-MTBs using four LLMs–Llama 3.2-vison: 90b, Claude 3.5 Sonnet, DeepSeek-R1, and OpenAI-o1 across five anonymized STS cases from the sarcoma ring trial. Each model was queried 21 times per case using a standardized prompt, and the responses were compared with human MTBs in terms of intra-model consistency, treatment recommendation alignment, alternative recommendations, and source citation.

**Results:**

LLMs demonstrated high inter-model and intra-model consistency in only 20% of cases, and their recommendations aligned with human consensus in only 20–60% of cases. The model with the highest concordance with the most common MTB recommendation, Claude 3.5 Sonnet, aligned with experts in only 60% of cases. Notably, the recommendations across MTBs were highly heterogenous, contextualizing the variable LLM performance. Discrepancies were particularly notable, where common human recommendations were often absent in LLM outputs. Additionally, the sources for the recommendation rationale of LLMs were clearly derived from the German S3 sarcoma guidelines in only 24.8% to 55.2% of the responses. LLMs occasionally suggested potentially harmful information were also observed in alternative recommendations.

**Conclusions:**

Despite the considerable heterogeneity observed in MTB recommendations, the significant discrepancies and potentially harmful recommendations highlight current AI tools’ limitations, underscoring that referral to high-volume sarcoma centers remains essential for optimal patient care. At the same time, LLMs could serve as an excellent tool to prepare for MDT discussions.

**Supplementary Information:**

The online version contains supplementary material available at 10.1007/s00432-025-06304-9.

## Introduction

Soft tissue sarcomas (STS) are a rare and heterogeneous group of tumors, comprising only about 1% of all adult malignancies (Gamboa et al. 2020). Therefore, most current sarcoma guidelines recommend that patients be diagnosed and treated at specialized sarcoma centers, where a multidisciplinary tumor board (MTB) or a multidisciplinary team (MDT) works collaboratively to guide treatment decisions (Dangoor et al. 2016; Gronchi et al. 2021; Casali et al. 2022; Tirotta et al. 2023; Jakob et al. 2023). The centralization of services and the implementation of MTB/MDT have significantly improved patient survival (Derbel et al. 2017; Blay et al. 2017, 2019, 2024; Kalaiselvan et al. 2019; Strönisch et al. 2023; Napolitano et al. 2024).

The sarcoma ring trial was the first cross-sectional study evaluating inter-center consensus in MTB recommendations for five standardized cases of localized STS. This prospective analysis across 21 referral sarcoma centers in Germany and Austria revealed a low agreement rate of 33.3% (Range: 9.3%-61.9%) in decision-making, demonstrating significant variability in management approaches among different centers (Roohani et al. 2025).

Artificial intelligence (AI), a branch of computer science that enables machines to perform tasks traditionally requiring human intelligence, is playing an increasingly significant role in cancer care (Perez-Lopez et al. 2024). AI tools, particularly generative AI (GenAI) like large language models (LLMs), have demonstrated significant potential in offering valuable information to guide treatment decisions for sarcoma patients (Li et al. 2024, 2025; Hernández‐Flores et al. 2025). While existing research has demonstrated the potential of LLMs in simulating MTB decision-making for sarcoma cases (Ammo et al. 2025), studies directly comparing LLM-generated suggestions with those from real MTBs are missing.

The purpose of this study is to investigate whether LLMs can effectively support STS-MTB. We compared the responses of four leading LLMs–Llama 3.2-vision:90b, Claude 3.5 Sonnet, DeepSeek-R1, OpenAI-o1- to five anonymized STS cases from the sarcoma ring trial, simulating the role of an MTB with the actual decisions of the MTBs from 21 German-speaking sarcoma centers.

## Methods

This study was performed in line with the principles of the Declaration of Helsinki. Approval was granted by the Ethics Committee of the Ruprecht-Karls-University Heidelberg (Medical Faculty Mannheim) (2025-524).

### Anonymized cases

The five anonymized patient cases of localized STS (two retroperitoneal, two extremity, and one trunk wall) described in the sarcoma ring trial (see supplementary file 1) were utilized in this study (Roohani et al. 2025). Four cases represented primary diagnosis and one involved recurrent disease. No metastatic disease was included. The cases included clinical information, pathological results and imaging studies (reports as well as two to three representative CT or MRI images per case). All text data were translated into English (see Supplementary file 1).

### Selection of models

We selected two closed-source (OpenAI-o1 and Claude 3.5 Sonnet) and two open-source (DeepSeek-R1 and Llama 3.2-vision:90b) LLMs to ensure a balanced evaluation. While closed-source models often benefit from vast private training resources and fine-tuning on biomedical literature, open-source models are cost-effective, transparent and accessible.

Since our tasks involve complex logical decision-making, we chose models with strong reasoning ability. With “Chain-of-Thoughts” (CoT) approach, OpenAI-o1 and DeepSeek-R1 demonstrated competitive performance in math and coding (DeepSeek-AI et al. 2025). Claude 3.5 Sonnet and Llama 3.2-vision:90b also show strong reasoning capabilities (Anthropic 2024; Meta AI 2024). Another criterion for model selection was the ability to interpret visual inputs, as we also presented CT or MRI images to the LLMs. While OpenAI-o1, Claude 3.5 Sonnet and Llama 3.2-vision:90b Vision are multimodal models with advanced visual reasoning, DeepSeek-R1 initially does not support image processing. We used the ChatboxAI platform (https://chatboxai.app), which adds a layer to extract information from scans, to enable it to generate responses using the interpreted visual content.

### Generation of LLM responses

To avoid bias, each LLM was queried using the same prompt and parameters on 14 February 2025. The query prompt was structured in accordance to the format in the sarcoma ring trial, mimicking a real MTB discussion (see Supplementary file 2). We assigned specific roles to LLMs within the prompt (“From now on, you are a panel of at least five medical experts specializing in sarcomas in Germany.”) and the roles as surgical oncologist, medical oncologist, radiation oncologist, radiologist and pathologist were described in detail. With the prompt, the output was set to have the identical format as in the sarcoma ring trial, including additional diagnostics (e.g. repeated imaging, biopsy), treatment recommendations with selections among modalities (surgery, radiotherapy, chemotherapy, etc.).

We accessed the models through different interfaces to generate responses. For Llama 3.2-vision:90b, we used its Application Programming Interfaces (API) on a locally installed model on a workstation equipped with dual GPUs (2× Nvidia RTX A6000, 48 GB GDDR6). Similarly, we programmatically interacted with Claude 3.5 Sonnet- 20241022 through its API. For these two models, the temperature parameter was set to zero to get deterministic results.

At the time of our study, we could not access DeepSeek-R1 and OpenAI-o1 through their APIs for different reasons: DeepSeek API charging function was deactivated and OpenAI was still rolling out the usage of o1-API. Hence, we decided to access OpenAI-o1 via its web-based interfaces (https://chatgpt.com), where users cannot configure the temperature setting, potentially introducing some variability in its responses. Since it is not possible to upload images on the web-based interface of DeepSeek-R1, we accessed DeepSeek-R1 via Chatbox (https://chatboxai.app), where the temperature was also set to zero.

To imitate the decision-making process of 21 sarcoma centers in sarcoma ring trial, each LLM was queried 21 times per case, each time in a new dialog to prevent any bias, learning, or memory effect from previous queries.

Given the absence of a definitive reference standard, we prespecified all agreement measures as descriptive concordance metrics. We did not perform hypothesis testing against a gold standard.

### Statistical methods

GraphPad Prism (version 10.3.1 for MacOS, GraphPad Software, Boston, Massachusetts USA, www.graphpad.com) was used for analysis and figure design. The intra-model agreement for each model was analyzed, as it was defined as the proportion of the most frequently recommended option across 21 rounds of inquiries. The alignment of the most common recommendations from human MTBs with those generated by the LLMs was assessed. In cases of disagreement, the proportion of the most preferred recommendations by MTBs present in the LLMs’ outputs was quantified, as was the proportion of the most frequent LLM recommendations found among human expert decisions. The most frequent advised alternative treatment strategies proposed by the LLMs were also analyzed.

We categorized the recommendations by combining pre- and postoperative treatment modalities into a single perioperative category, excluding hyperthermia due to its regional nature and availability. A comparison was also conducted between the alignment of the predominant grouped recommendations by LLMs and those by human centers.

A comprehensive analysis of the rationales underlying the recommendations provided in the four models was also conducted. The sources were methodically classified into the following categories: (1) recommendations exclusively based on the German S3 guidelines (German Cancer Society 2022); (2) recommendations based on the German S3 guidelines in combination with other guidelines, clinical trials, or studies; (3) recommendations based on other guidelines, e.g., National Comprehensive Cancer Network (NCCN) or European Society for Medical Oncology (ESMO) guidelines for soft tissue sarcoma. The recommendations can be made in conjunction with data from other trials or studies but without the German S3 guidelines; (4) recommendations based on clinical trials and/or studies, without any clinical guidelines; (5) recommendations that were not based on any guidelines, clinical trial data, or published studies.

## Results

Each case was subjected to each LLM for 21 times, Llama 3.2-vision:90b generated a total of 6 recommendations, Claude 3.5 Sonnet generated 5 recommendations, DeepSeek-R1 and OpenAI-o1 both generated 23 recommendations (Table 1). The inter-institutional agreements of 21 sarcoma centers and the intra-model agreements of the LLMs are displayed in Table 2. The model that demonstrated the highest internal consistency was Claude 3.5 Sonnet, which generated 100% consistent results in 21 repeated inquiries for each case. It was followed by Llama 3.2-vision:90b, which exhibited an intra-model agreement of 100% for 4 cases and 61.9% for one case. The median intra-model consistency of Deepseek-R1 and OpenAI-o1 is comparable, with both demonstrating a median agreement of 47.6% (Ranges: 28.6%–100% for Deepseek-R1, and 38.1%–100% for OpenAI-o1).

**Table 1:** Number of proposed treatment options identified by MDTs and the LLM systems

Case no.	Human MDT	Llama 3.2-vision:90b	Claude 3.5 Sonnet	DeepSeek-R1	OpenAI-o1	All 4 LLMs
1	15	1	1	4	7	8
2	9	2	1	5	6	8
3	11	1	1	10	5	14
4	4	1	1	1	1	1
5	18	1	1	3	4	5

**Table 2:** Inter-institutional and Intra-LLM agreement for 5 cases

Case no.	Human MDT (%)	Llama 3.2-vision:90b (%)	Claude 3.5 Sonnet (%)	DeepSeek-R1 (%)	OpenAI-o1 (%)
1	14.3	100	100	47.6	38.1
2	61.9	100	61.9	28.6	42.9
3	33.3	100	100	42.9	66.7
4	52.4	100	100	100	100
5	9.3	100	100	90.5	47.6

### Analysis of recommendations by LLMs

#### Case 1: localized dedifferentiated liposarcoma (G2) in the right groin

**Fig. 1:**
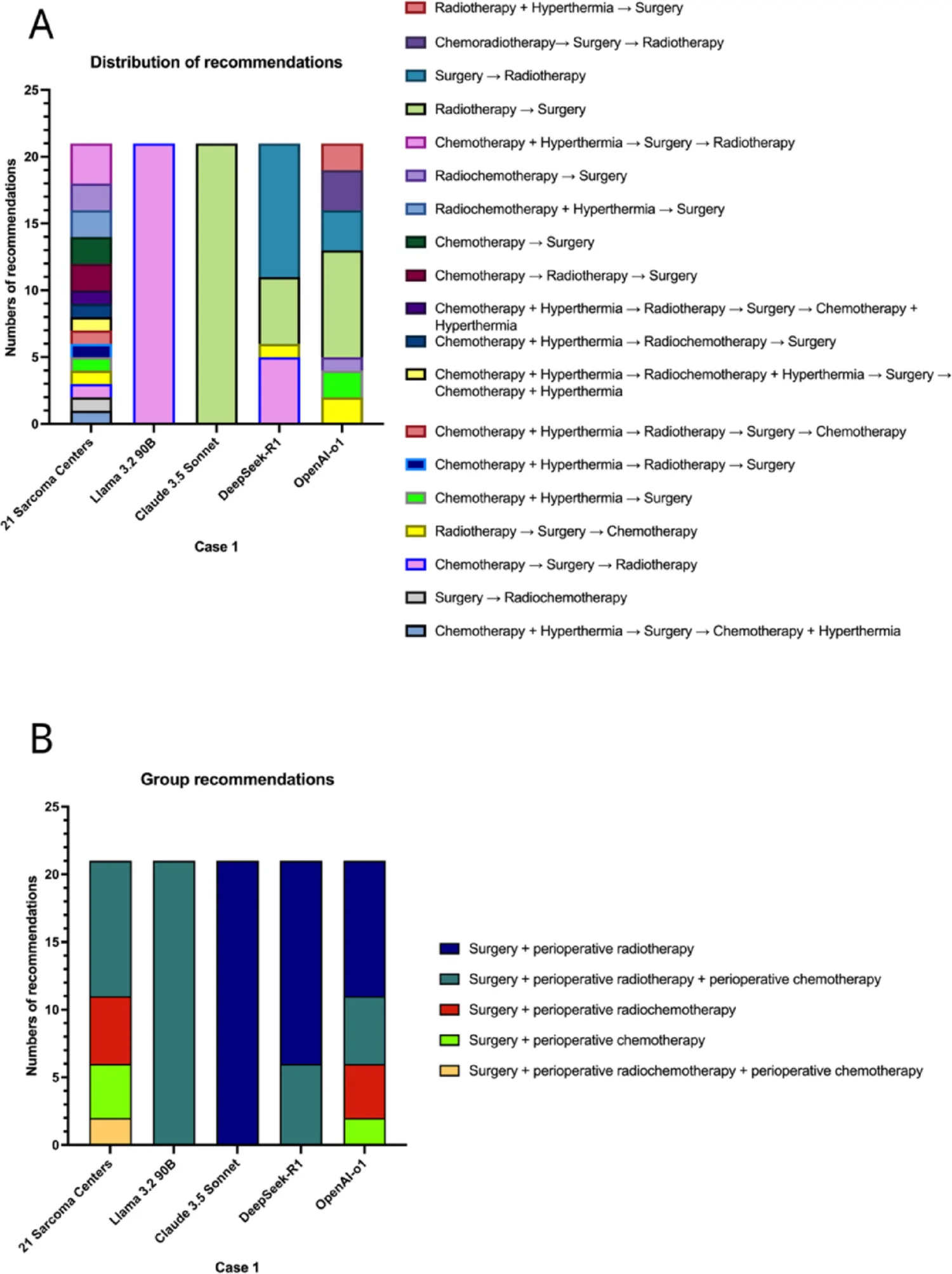
Analysis of recommendations of 21 sarcoma MTBs and 4 LLMs for case 1: localized dedifferentiated liposarcoma (G2) in the right groin. **A** Detailed treatment recommendations are outlined, including specific therapy modalities and sequences. **B** Grouped recommendations are summarized by combining preoperative and postoperative treatment modalities into “perioperative treatment modalities”

In regard to Case 1, four LLMs generated a total of eight distinct recommendations (see Fig. 1A). Llama 3.2-vision:90b and Claude 3.5 Sonnet demonstrated the highest intra-model consistency in their responses, providing the same recommendation in 21 repeated inquiries. Llama 3.2-vision:90b recommended neoadjuvant chemotherapy, followed by wide surgical resection and adjuvant radiotherapy. This recommendation was endorsed by only one center (4.8%) in the human MBT decisions. Claude 3.5 Sonnet suggested neoadjuvant radiotherapy, followed by wide surgical resection, which was not supported by any center. DeepSeek-R1 provided a total of three distinct recommendations, the most common of which was adjuvant radiotherapy after resection (10/21, 47.6%), a recommendation that was not made by any center either. Of the seven different recommendations given by OpenAI-o1, the most common was surgery after neoadjuvant radiotherapy (8/21, 38.1%), which was also not recommended by any MTB. In the sarcoma ring trial, the most common recommendation with an agreement percentage of 14.3% from three centers was regional hyperthermia with chemotherapy, followed by surgical resection and postoperative radiotherapy. This recommendation had never been suggested by any LLM.

In grouped analysis, the most frequently recommended treatment approach by 10 MTBs was surgery with perioperative radiotherapy and perioperative chemotherapy with or without regional hyperthermia (47.6%), which is also the predominant suggestion from Llama 3.2-vision: 90b (see Fig. 1B). However, the favored grouped recommendation by other 3 models was surgery with perioperative radiotherapy (Claude 3.5 sonnet: 100%, DeepSeek-R1: 76.2%, and OpenAI-o1: 47.6%), a combination not suggested by any MTB in the sarcoma ring trial.

In summary, the results reveal a striking divergence between LLM-generated recommendations and those made by human MBTs for case 1, despite the high intra-model consistency demonstrated by Llama 3.2-vision:90b and Claude 3.5 Sonnet.

#### Case 2: localized myxoid liposarcoma (G3) in the right shoulder blade

**Fig. 2:**
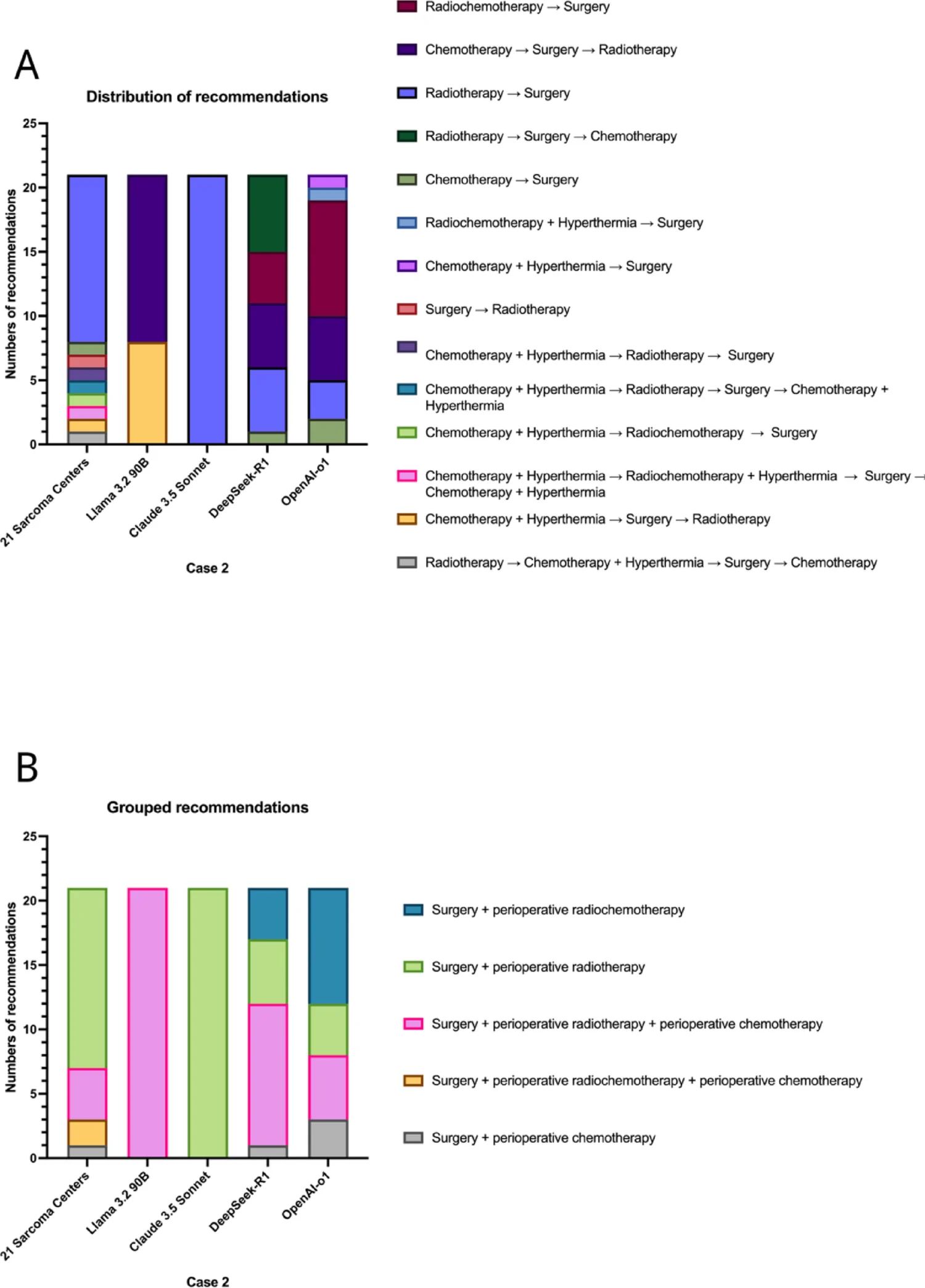
Analysis of recommendations of 21 sarcoma MTBs and 4 LLMs for case 2: localized myxoid liposarcoma (G3) in the right shoulder blade. **A** Detailed treatment recommendations are outlined, including specific therapy modalities and sequences. **B** Grouped recommendations are summarized by combining preoperative and postoperative treatment modalities into “perioperative treatment modalities”

For Case 2, the LLMs generated a total of eight distinct recommendations (see Fig. 2A). The analysis revealed that the Claude 3.5 Sonnet exhibited the most consistency in its responses, offering a unique recommendation for neoadjuvant radiotherapy followed by wide resection, which was the most common one made by 13 centers (61.9%) in the sarcoma ring trial. In contrast, this recommendation accounts for 23.8% (5/21) of the DeepSeek-R1 and 14.3% (3/21) of the OpenAI-o1. Notably, Llama 3.2-vision:90b did not recommend this, whereas all its recommendations are approaches based on neoadjuvant chemotherapy. A total of 61.9% (13/21) of its recommendations entail neoadjuvant chemotherapy followed by surgery, while the remaining 33.3% (8/21) of recommendations include preoperative hyperthermia in addition to the former. Notably, none of the 21 sarcoma centers offered the former recommendation of Llama 3.2-vision:90b, and only one center provided the latter. The most frequent recommendations provided by DeepSeek-R1 and OpenAI-o1 are, respectively, neoadjuvant radiotherapy followed by surgical resection and adjuvant chemotherapy (6/21, 28.6%) and surgery after neoadjuvant radiochemotherapy (9/21, 42.9%). Notably, these recommendations are not among the 21 center recommendations. To summarize, Claude 3.5 Sonnet surpassed other three LLMs regarding alignment with human expertise in case 2. (Claude 3.5 Sonnet > DeepSeek R1 > openAI o1 > Llama 3.2 -vision).

In grouped analysis, the predominant grouped recommendation by 14 MTBs (66.7%) was surgery with perioperative radiotherapy, which is also the unique treatment modality provided by Claude 3.5 sonnet (see Fig. 2B). The favored grouped recommendation by Llama 3.2-vision: 90b (100%) and DeepSeek-R1 (52.4%) was surgery with perioperative chemotherapy and perioperative radiotherapy, a combination suggested by 4 MTBs (19.0%) in the sarcoma ring trial. However, the most common recommendation by OpenAI-o1 is surgery with perioperative radiochemotherapy (9/21, 42.9%), which was not suggested by any human center.

#### Case 3: locally recurrent dedifferentiated liposarcoma (G2) in the right retroperitoneum

**Fig. 3:**
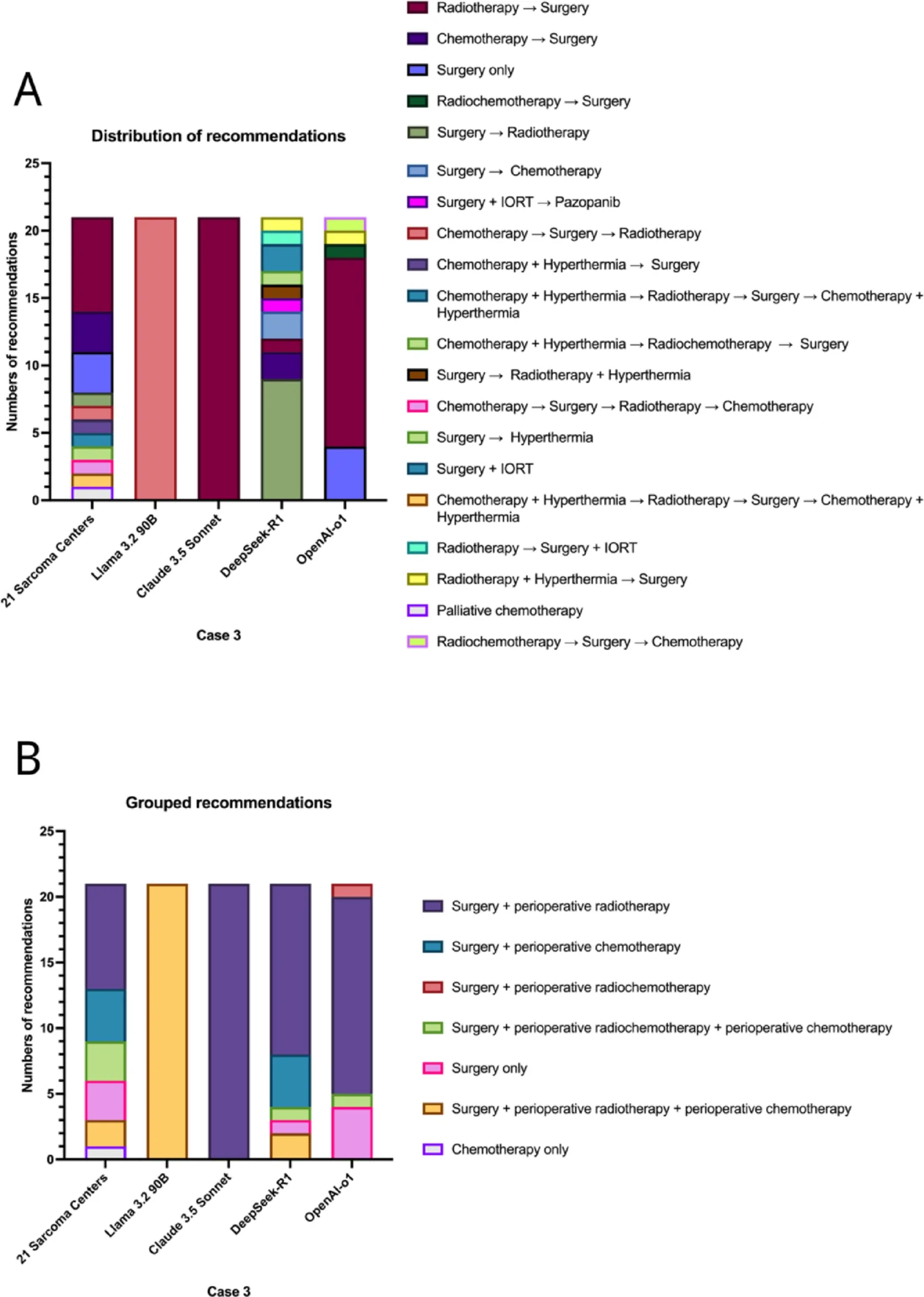
Analysis of recommendations of 21 sarcoma MTBs and 4 LLMs for case 3: locally recurrent dedifferentiated liposarcoma (G2) in the right retroperitoneum. **A** Detailed treatment recommendations are outlined, including specific therapy modalities and sequences. **B** Grouped recommendations are summarized by combining preoperative and postoperative treatment modalities into “perioperative treatment modalities”

In Case 3, four LLMs yielded a total of 14 distinct recommendations (see Fig. 3A). Both Llama 3.2-vision:90b and Claude 3.5 Sonnet exhibited the most intra-model robustness in their responses, providing the same recommendation in each of the 21 repeated inquiries. Llama 3.2-vision:90b recommended neoadjuvant chemotherapy, followed by surgical resection and adjuvant radiotherapy. This recommendation was endorsed by only one center (4.8%) in the ring trial. Claude 3.5 Sonnet suggested the sequence of neoadjuvant radiotherapy and surgery which is the most prevalent recommendation among the 21 sarcoma centers (7/21, 33.3%). This therapeutic sequence is also the most common recommendation by OpenAI-o1 (14/21, 66.7%), but only one by DeepSeek-R1 (4.8%). The predominant recommendation for DeepSeek-R1 is surgical resection with postoperative radiotherapy (9/21), which is exclusively made by a single sarcoma center (4.8%).

Generally, Claude 3.5 Sonnet demonstrated the strongest alignment with MTBs. (Claude 3.5 Sonnet > OpenAI-o1 > DeepSeek-R1 = Llama3.2-vision).

In grouped analysis, the predominant grouped recommendation by 8 MTBs (38.1%) was surgery with perioperative radiotherapy, which is also the favored treatment modality provided by Claude 3.5 sonnet (100%), DeepSeek-R1 (61.9%) and OpenAI-o1 (71.4%) (see Fig. 3B). The favored grouped recommendation by Llama 3.2-vision was surgery with perioperative radiotherapy and perioperative chemotherapy, a combination suggested by 2 human centers (9.5%) in the sarcoma ring trial. If perioperative radiochemotherapy, radiochemotherapy plus chemotherapy and radiotherapy plus chemotherapy are further combined into one category, the results of most group recommendations (Table 3) are similar to those described above, surgery with perioperative radiotherapy is also the favored recommendations by human MTBs, Claude 3.5 sonnet, DeepSeek-R1 and OpenAI-o1.

**Table 3:** Numbers of most grouped recommendations of 21 centers and 4 LLMs for case 3

Recommendations	Human MDT	Llama 3.2-vision:90b	Claude 3.5 Sonnet	DeepSeek-R1	OpenAI-o1
Surgery only	3 (14.3%)	0 (0%)	0 (0%)	1 (4.8%)	4 (19.0%)
Surgery + RT	8 (38.1%)	0 (0%)	21 (100%)	13 (62.9%)	15 (71.4%)
Surgery + CT	4 (19.0%)	0 (0%)	0 (0%)	4 (19.0%)	0 (0%)
Surgery + RT + CT	5 (23.8%)	21 (100%)	0 (0%)	3 (14.3%)	2 (9.5%)
CT only	1 (4.8%)	0 (0%)	0 (0%)	0 (0%)	0 (0%)

RT, radiotherapy; CT, chemotherapy

#### Case 4: well‐differentiated liposarcoma (G1) in the right retroperitoneum

**Fig. 4:**
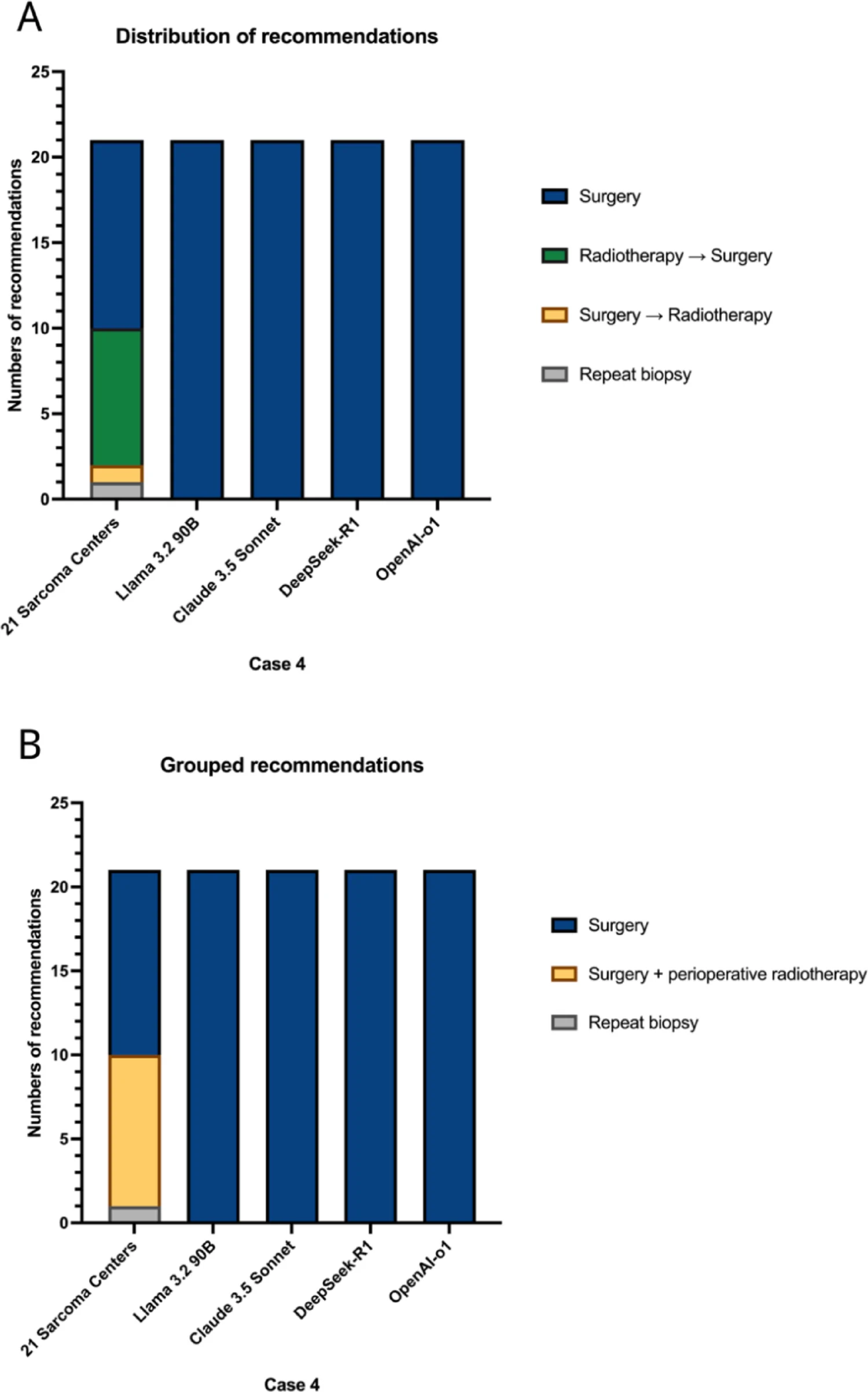
Analysis of recommendations of 21 sarcoma MTBs and 4 LLMs for case 4: well-differentiated liposarcoma (G1) in the right retroperitoneum. **A** Detailed treatment recommendations are outlined, including specific therapy modalities and sequences. **B** Grouped recommendations are summarized by combining preoperative and postoperative treatment modalities into “perioperative treatment modalities”

In Case 4, the LLMs demonstrated remarkable inter-model and intra-model consistency (see Fig. 4A, B), with each model independently recommending surgical resection without any adjuvant therapy in all 21 inquiries (100%). This recommendation was also the most prevalent (11/21, 52.4%) in 21 sarcoma centers in the ring trial.

#### Case 5: localized pleomorphic rhabdomyosarcoma (G3) in the right popliteal fossa

**Fig. 5:**
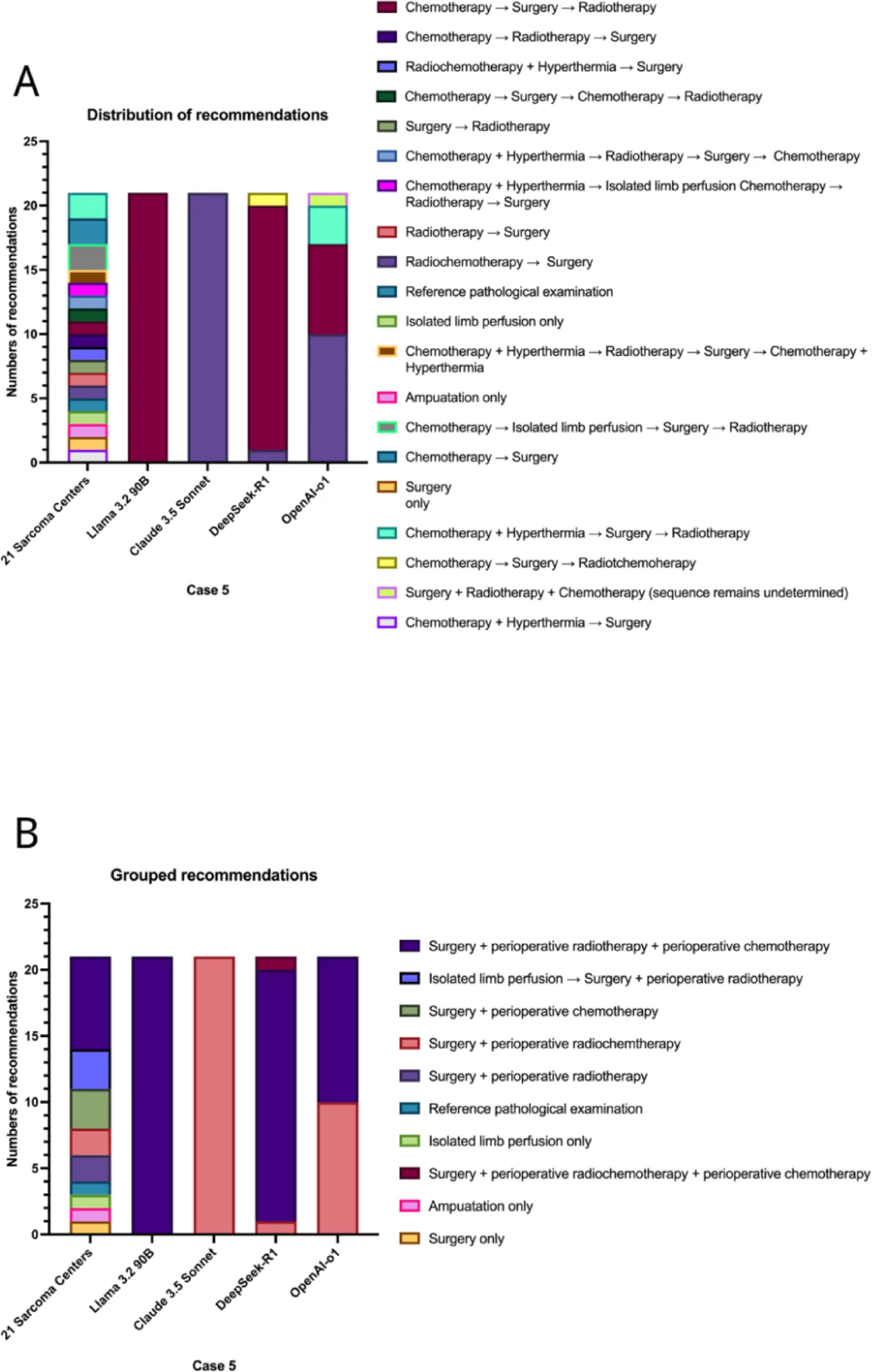
Analysis of recommendations of 21 sarcoma MTBs and 4 LLMs for case 5: localized pleomorphic rhabdomyosarcoma (G3) in the right popliteal fossa. **A** Detailed treatment recommendations are outlined, including specific therapy modalities and sequences. **B** Grouped recommendations are summarized by combining preoperative and postoperative treatment modalities into “perioperative treatment modalities”

In Case 5, four LLMs yielded a total of five different recommendations (see Fig. 5A). Both Llama 3.2-vision:90b and Claude 3.5 Sonnet showed the most intra-model robustness in their responses, providing the same recommendation in each of the 21 repeated inquiries. Llama 3.2-vision:90b recommended a treatment sequence of neoadjuvant chemotherapy followed by surgical resection and adjuvant radiotherapy, which is also the most common recommendation (19/21, 90.5%) by DeepSeek-R1. Claude 3.5 Sonnet gave a unanimous suggestion of radiochemotherapy followed by surgical resection, which is also the prevalent recommendation (10/21, 47.6%) by OpenAI-o1. However, these frequently recommended approaches across four large language models (LLMs) were only suggested once in the opinions of all the MTB with human experts (4.8%).

Actually, the sarcoma ring trial revealed that the recommendations for Case 5 from the 21 sarcoma centers exhibited significant variability, with the highest inter-center agreement of 9.5% (2/21). This finding indicates a lack of consensus among the centers. The highest level of agreement was observed across three treatment strategies: preoperative chemotherapy combined with regional hyperthermia followed by surgery and postoperative radiotherapy; preoperative chemotherapy combined with surgery; and preoperative chemotherapy combined with isolated limb perfusion (ILP), then surgery and postoperative radiotherapy. Notably, the first two common treatment strategies are not recommended by any LLM, while 14.3% (3/21) of OpenAI-o1 responses advised that preoperative chemotherapy combined with hyperthermia, then surgery and postoperative radiotherapy for this case.

In the grouped analysis, the most common grouped recommendation suggested by 7 MTBs (33.3%) was surgery with perioperative chemotherapy and perioperative radiotherapy, which was also the dominant responses by Llama 3.2-vision: 90b (100%), DeepSeek-R1 (90.5%) and OpenAI-o1 (52.4%) (see Fig. 5B). Claude 3.5 sonnet’s unique response (surgery with perioperative radiochemotherapy) was suggested by 2 MTBs in the ring trial.

#### Overall alignment between LLMs’ recommendations and human MTBs

In the context of the most common recommendation from the 21 MTBs being used as a benchmark, Claude 3.5 Sonnet demonstrated the most favorable performance among all LLMs. Specifically, 60% of its top recommendations (in Cases 2, 3, and 4) showed alignment with those of the human MTB. However, Claude 3.5 Sonnet demonstrated very poor performance in the other two cases, with its most common recommendation absent among human recommendations in Case 1 and supported by only one MTB in Case 5. The remaining three models (Llama 3.2-vision:90b, DeepSeek-R1, and OpenAI-o1) exhibited a relatively lower degree of alignment with the human MTB, with their most prevalent recommendations aligning with those of the human experts in only 20%, 20%, and 40% of cases, respectively.

Case-by-case analysis demonstrated that all LLMs performed best with the optimal level of agreement (100% intra-model agreement) in Case 4. Each model independently recommended surgical resection without any adjuvant therapy in all 21 inquiries (100%). This treatment strategy was recommended by more than half of the 21 sarcoma centers (11 out of 21, 52.4%) that participated in the sarcoma ring trial. The most significant discrepancy between the performance of LLMs and that of human experts was observed in Case 1, where the predominant suggestion from human experts (regional hyperthermia with chemotherapy, followed by surgical resection and postoperative radiotherapy) was not provided by any LLMs in 21 rounds of inquiries. Meanwhile, in Case 1, the most common opinion among LLM models was rarely endorsed by human experts too. The sole recommendation of Llama 3.2-vision:90b was suggested by only one center (4.8%) in the ring trial, and the predominant recommendations of the remaining three models had never been proposed by any human MTB. A similar phenomenon can be observed in Cases 2 and 5. In Case 2, with the exception of Claude 3.5 Sonnet, whose most common recommendation aligns with the human MTB, the most common recommendations of the other three models are not present in the that of the human MTBs. In Case 5, the most common suggestions of the four models each align with 4.8% of (1/21) human MTB teams.

### Source of recommendation rationale

**Fig. 6:**
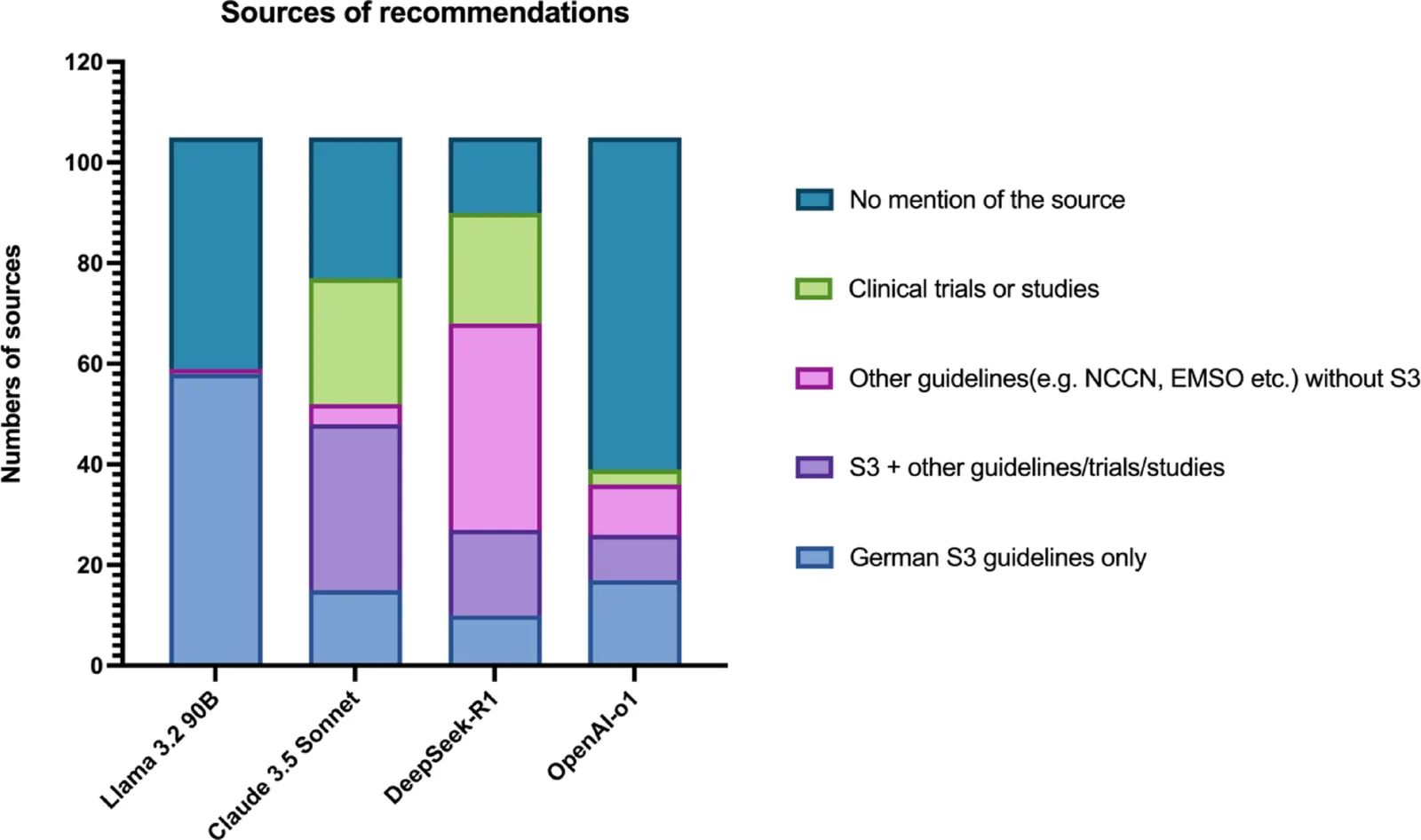
Rationale sources of recommendations of 4 LLMs. The sources were methodically classified into the 5 categories: (1) recommendations exclusively based on the German S3 guidelines; (2) recommendations based on the German S3 guidelines in combination with other guidelines, clinical trials, or studies; (3) recommendations based on other guidelines, e.g., National Comprehensive Cancer Network (NCCN) or European Society for Medical Oncology (ESMO) guidelines for soft tissue sarcoma. The recommendations can be made in conjunction with data from other trials or studies but without the German S3 guidelines; (4) recommendations based on clinical trials and/or studies, without any clinical guidelines; (5) recommendations that did not based on any guidelines, clinical trial data, or published studies

The results of the analysis on the rationale behind recommendations across 105 inquiries per model are presented in Fig. 6. The proportions of recommendations derived exclusively or partially from the German S3 guidelines, in descending order, were as follows: Llama 3.2-vision:90b (58/105, 55.2%), Claude 3.5 Sonnet (48/10, 45.7%), DeepSeek-R1 (27/105, 25.7%), and OpenAI-o1 (26/105, 24.8%). Notably, Llama 3.2-vision:90b was the only model for which all S3 guideline–related rationales were derived solely from the German S3 guidelines, without reliance on other guidelines, trials, or studies; however, it also provided 43.8% (46/105) of recommendations without citing any source. Deepseek-R1 showed the lowest proportion of recommendations based solely on the German S3 guidelines (15/105, 14.3%), while concurrently having the lowest proportion lacking any cited source (28/105, 26.7%). OpenAI-o1 exhibited the highest proportion of recommendations without any cited source, reaching 62.9% (66/105), the greatest among all models. In addition, DeepSeek-R1 exhibited the highest proportion of recommendations per guidelines other than the S3 guidelines, at 39.0% (41/105), which is significantly higher than those observed for the other three models—9.5% for OpenAI-o1, 3.8% (4/105) for Claude 3.5 Sonnet, and 1.0% (1/105) for Llama 3.2-vision:90b. Regarding recommendations based on data of other clinical trials and studies that are not guidelines, Claude 3.5 Sonnet and DeepSeek-R1 accounted for 23.8% (25/105) and 21.0% (22/105), respectively; in contrast, Llama 3.2-vision:90b did not use such sources, and OpenAI-o1 accounted for only 2.9% (3/105).

### Analysis of recommended best alternative therapy by LLMs

For Case 1, surgical resection with adjuvant radiochemotherapy was the predominant alternative recommendation by Llama 3.2-vision:90b (21/21, 100%). Surgical resection with adjuvant radiotherapy was the overwhelming recommendation, particularly in cases where the margin was close or positive, by Claude 3.5 Sonnet (21/21, 100%). This consensus was also the prevailing one by OpenAI-o1 (10/21, 47.6%). In contrast, DeepSeek-R1 primarily suggested neoadjuvant radiotherapy followed by surgery (6/21, 28.6%). Notably, neither approach was endorsed by any of centers participating in the ring trial.

For Case 2, surgical resection with adjuvant radiotherapy remained the top alternative recommendation from Llama 3.2-vision:90b (21/21, 100%), Claude 3.5 Sonnet (21/21, 100%), and OpenAI-o1 (8/21, 38.1%), aligning with one MTB’s recommendation in the ring trial. Meanwhile, DeepSeek-R1 most frequently proposed neoadjuvant radiotherapy followed by surgery (4/21, 19.0%), consistent with the predominant approach among human MTBs.

For Case 3, Llama 3.2-vision:90b predominantly recommended enrollment in an MDM2/CDK4 inhibitor trial (20/21, 95.2%) as an alternative approach. Claude 3.5 Sonnet (11/21, 53.8%) and OpenAI-o1 (15/21, 71.4%) favored surgical resection alone, an approach endorsed by three sarcoma centers in the ring trial. DeepSeek-R1's most frequent alternative recommendation was radiotherapy (12/21, 57.1%).

For Case 4, Llama 3.2-vision:90b primarily recommended radiotherapy (20/21, 95.2%), while Claude 3.5 Sonnet (20/21, 95.2%), DeepSeek-R1 (21/21, 100%), and OpenAI-o1 (19/21, 90.5%) all favored active surveillance or observation.

For Case 5, Llama 3.2-vision:90b and Claude 3.5 Sonnet exclusively recommended surgery with postoperative radiotherapy (21/21, 100%), while OpenAI-o1 most frequently suggested surgery with postoperative radiochemotherapy (19/21, 90.5%). In contrast, DeepSeek-R1 recommended amputation with an intra-model agreement of 85.7% (18/21).

## Discussion

To the best of our knowledge, this is the first study to employ four widely used LLMs–Llama 3.2-vision:90b, Claude 3.5 Sonnet, DeepSeek-R1, and OpenAI-o1, to simulate STS-MTB and to compare their decision-making capabilities for real STS cases with 21 German-speaking sarcoma MTBs. The findings of this study revealed significant discrepancies between the recommendation tendencies of LLMs and human MTBs for complex STS cases. First, the best-performing model, Claude 3.5 Sonnet, aligned with 60% (3/5) of the human experts’ consensus. However, in the two remaining cases, its performance was suboptimal. Second, a case-by-case analysis highlights that although all LLMs aligned with the predominant human expert opinion (surgery only) in Case 4, they failed to align with human consensus in some other instances. For example, in Case 1, the most common recommendation made by human MTBs was never suggested by any LLM. However, these frequently recommended approaches across four large language models (LLMs) were rarely suggested in the opinions of all the MTB with human experts (4.8%). (e.g. Case 5).

The present study is distinguished from previous research on LLMs performing tasks related to sarcoma care in two significant ways. Firstly, it employed anonymized real cases comprising medical history, pathology, and imaging to validate the capability of LLMs to simulate sarcoma MTB. These cases are all challenging, as evidenced by the median inter-center agreement of only 33.3% in the Ring Trial (Roohani et al. 2025). Previous studies on LLM's response to sarcoma-related questions have used simple, straightforward questions, which are less challenging (Valentini et al. 2024; Li et al. 2024). However, real-world cases are often highly complex, even minor pitfalls and subtle ambiguities in the questions could lead the model to provide incorrect answers (Li et al. 2025). Even with more advanced, reasoning models, the consistency of LLM-generated recommendations still differed greatly from those of MDTs in our study. Secondly, all the responses by LLMs were compared with the recommendations of real MTB teams from the 21 referral sarcoma centers. These centers included 20 German centers all certified by the German Cancer Society (DKG) and one Austrian center. The recommendations of these centers can reflect the current STS treatment concepts in Germany. However, in previous studies, LLMs' responses to STS-related questions were evaluated by a few sarcoma experts in a single center using one of two approaches: either by assessing with existing sarcoma guidelines (Valentini et al. 2024; Li et al. 2024, 2025) or through a single-institutional MTB approach (Ammo et al. 2025).

In previous research on other tumors, LLMs had demonstrated significant concordance with the real MTB consensus (Horesh et al. 2025; Chatziisaak et al. 2025; Karabuğa et al. 2025). However, LLMs failed to accurately replicate specific treatment plans, especially chemotherapy protocols and follow-up procedures, even in some straightforward cases, renders it unsuitable for direct clinical decision-making (Aghamaliyev et al. 2024). Similarly, the results of this study indicate a low agreement between LLMs and MTBs, which primarily attributed to the rarity of STS cases and clinical data, as well as the varying treatment standards among real-world sarcoma centers. As outlined in the discussion section of the ring trial, the low inter-center agreement highlights the scarcity of clinical data and the influence of center-specific conventions. Given this context, it is unsurprising that LLMs also exhibit considerable variability and often do not cite specific sources or trials to support their treatment recommendations. Moreover, the absence of a clear "ground truth" or unequivocal clinical standard in all cases further limits the ability to meaningfully evaluate LLM outputs, as there may be no single correct answer against which to benchmark them. The variability in recommendations from the 21 centers in the Ring trial results highlights the inconsistency among current STS practices across different sarcoma centers. Notably, in Case 4, despite being regarded as less complex compared to the other four cases, expert recommendations still showed variability, while all LLMs unanimously recommended surgery only. This inconsistency arises not only from the rarity of STS but also from the distinct historical conventions and practices of individual centers. This inconsistency limits the interpretability of LLM performance, yet at the same time reflects the real-world difficulties faced by clinicians when managing complex sarcoma cases. Furthermore, the absence of a definitive “ground truth” makes the evaluation of LLM reliability nearly impossible. This heterogeneity highlights the fact that benchmarking AI against human experts in rare conditions like sarcoma is inherently limited because there is no single correct answer. In addition, not only the objective level of competence, but also the subjective perception of the treating physician plays a significant role in the treatment satisfaction and treatment outcome. The public views physicians as more trustworthy sources of medical information than AI tools (Reis et al. 2024). While LLMs shows potential as a supportive tool for managing complex cancer cases and may serve as a foundation for further discussion in MTBs, it is insufficient as a standalone decision-maker in clinical practice (Karabuğa et al. 2025).

It is noteworthy that, although the role we assigned to LLMs in the prompt was a panel of medical experts specializing in sarcomas in Germany, according to the source analysis of the recommendation provided by LLMs, only Llama 3.2-vision:90b provided more than half of the recommendation, with rationales coming from the German S3 guidelines. Some LLMs like DeepSeek-R1 tended to use the other guidelines more frequently than the German guidelines (39.0% vs. 25.7%). Despite most of sarcoma guidelines containing similar key recommendations, subtle differences do exist. For instance, tumor necrosis factor-α (TNF-α), a frequently used agent in ILP and a common recommendation in European guidelines, has not received approval in the United States (Jakob and Hohenberger 2016). Therefore, using other guidelines, as well as differences in potentially available medications or treatments in different countries or regions, may influence the decision-making of LLMs.

In addition, previous research has found that LLMs often generate potentially harmful or even life-threatening responses in rare diseases like sarcoma (Li et al. 2024, 2025). This phenomenon was also observed in this study, particularly in the alternative recommendations given by LLMs. For instance, for a patient with retroperitoneal WDLPS with increasing digestive problems and gastric emptying disorders in Case 4, the overwhelming alternative recommendations for Claude 3.5 Sonnet, DeepSeek-R1, and OpenAI-o1 are active surveillance or observation, which is clearly inaccurate. In addition, DeepSeek-R1 recommended amputation in Case 5, with an intra-model agreement of 85.7% (18/21). This recommendation contradicts the statement that the indication for amputation should be made after exploring all limb-preserving options, as outlined in the German S3 guidelines. These erroneous suggestions have compromised the credibility of LLMs, underscoring the current limitations of generative AI models which are not specifically trained on medical or oncology datasets and are expected to struggle with more complex medical decision-making tasks (Hernández‐Flores et al. 2025). Implementing a Retrieval-augmented Generation (RAG) approach could address these problems by providing external data sources, such as the German S3 guidelines, to enhance LLM outputs. This approach would improve both source transparency and regional adaptability in generating therapy recommendations (Li et al. 2025).

Our study has some limitations. First, although the cases were originally discussed in German during the sarcoma ring trial, they were translated into English before being presented to the LLMs. While we tried our best to maintain accuracy, some nuances might have been lost in translation. Second, continuous scans of all radiological sequences were provided in the Sarcoma Ring Trial. Due to technical limitations, only selected, representative slides which were published as supplementary materials were submitted to LLMs in our study. Unlike MTBs, which could assess full radiologic datasets, LLMs were limited to representative images, handicapping their surgical decision-making capacity. Third, the small sample size of five cases hinders the ability to conduct a thorough statistical analysis of the degree of agreement among human MTBs and LLMs. Because the Ring Trial dataset only contained challenging cases, less controversial cases with full agreement were not available. Future work should include such cases to better benchmark LLM performance. Fourth, the temperature parameter can't be set to be zero for OpenAI-o1, even with API (De Winter et al. 2024). Temperature is critical to ensuring the randomness of the generation process. Higher temperatures may result in a greater variety, which has the potential to lead to inconsistent results among different inquiries (Peeperkorn et al. 2024). Moreover, the “rationales” provided by the LLMs contain very limited information, so that it was impossible to double-check whether the cited sources were correct or hallucinated.

## Conclusions

This study presents a pioneering effort to benchmark the decision-making processes of four advanced LLMs by comparing them to the recommendations of MTBs from 21 sarcoma centers in Germany and Austria. The results of this study revealed that the overall alignment with human expert recommendations was suboptimal, with the best-performing model, Claude 3.5 Sonnet, matching human consensus in only 60% of cases. The analysis identified significant discrepancies between LLMs and human experts and some potentially harmful alternative recommendations of LLMs. These findings highlight the present limitations of generative AI models in managing the intricacies of STS management. Furthermore, the variability in guideline adherence and source utilization across models underscores the importance of integrating region-specific clinical standards in AI training. While LLMs could serve as a valuable tool to prepare for MDT discussions by providing preliminary information and potential treatment options for consideration, referral to high-volume sarcoma centers with specialized expertise and a multidisciplinary approach remains the only approach proven to benefit patients with suspected soft tissue sarcoma currently.

## Supplementary Information

Below is the link to the electronic supplementary material.

Supplementary Material 1

Supplementary Material 2

## Data Availability

No datasets were generated or analysed during the current study.

## References

[CR1] Aghamaliyev U, Karimbayli J, Giessen-Jung C et al (2024) ChatGPT’s gastrointestinal tumor board tango: a limping dance partner? Eur J Cancer 205:114100. 10.1016/j.ejca.2024.114100 38729055 10.1016/j.ejca.2024.114100

[CR2] Ammo T, Guillaume VGJ, Hofmann UK et al (2025) Evaluating ChatGPT-4o as a decision support tool in multidisciplinary sarcoma tumor boards: heterogeneous performance across various specialties. Front Oncol 14:1526288. 10.3389/fonc.2024.1526288 39896191 10.3389/fonc.2024.1526288 PMC11782276

[CR3] Anthropic (2024) Introducing Claude 3.5 Sonnet. https://www.anthropic.com/news/claude-3-5-sonnet. Accessed 21 Mar 2025

[CR4] Blay J-Y, Honoré C, Stoeckle E et al (2019) Surgery in reference centers improves survival of sarcoma patients: a nationwide study. Ann Oncol 30:1143–1153. 10.1093/annonc/mdz124 31081028 10.1093/annonc/mdz124 PMC6637376

[CR5] Blay JY, Penel N, Valentin T et al (2024) Improved nationwide survival of sarcoma patients with a network of reference centers. Ann Oncol 35:351–363. 10.1016/j.annonc.2024.01.001 38246351 10.1016/j.annonc.2024.01.001

[CR6] Blay J-Y, Soibinet P, Penel N et al (2017) Improved survival using specialized multidisciplinary board in sarcoma patients. Ann Oncol 28:2852–2859. 10.1093/annonc/mdx484 29117335 10.1093/annonc/mdx484 PMC5834019

[CR7] Casali PG, Blay JY, Abecassis N et al (2022) Gastrointestinal stromal tumours: ESMO–EURACAN–GENTURIS clinical practice guidelines for diagnosis, treatment and follow-up. Ann Oncol 33:20–33. 10.1016/j.annonc.2021.09.005 34560242 10.1016/j.annonc.2021.09.005

[CR8] Chatziisaak D, Burri P, Sparn M et al (2025) Concordance of ChatGPT artificial intelligence decision-making in colorectal cancer multidisciplinary meetings: retrospective study. BJS Open 9:zraf040. 10.1093/bjsopen/zraf040 40331891 10.1093/bjsopen/zraf040 PMC12056934

[CR9] Dangoor A, Seddon B, Gerrand C et al (2016) UK guidelines for the management of soft tissue sarcomas. Clin Sarcoma Res 6:20. 10.1186/s13569-016-0060-4 27891213 10.1186/s13569-016-0060-4 PMC5109663

[CR10] De Winter JCF, Dodou D, Eisma YB (2024) System 2 thinking in OpenAI’s o1-preview model: near-perfect performance on a mathematics exam. Computers 13:278. 10.3390/computers13110278

[CR11] DeepSeek-AI, Guo D, Yang D et al (2025) DeepSeek-R1: incentivizing reasoning capability in LLMs via reinforcement learning. http://arxiv.org/abs/2501.12948. Accessed 11 Feb 2025

[CR12] Derbel O, Heudel PE, Cropet C et al (2017) Survival impact of centralization and clinical guidelines for soft tissue sarcoma (a prospective and exhaustive population-based cohort). PLoS ONE 12:e0158406. 10.1371/journal.pone.0158406 28158190 10.1371/journal.pone.0158406 PMC5291382

[CR13] Gamboa AC, Gronchi A, Cardona K (2020) Soft-tissue sarcoma in adults: an update on the current state of histiotype-specific management in an era of personalized medicine. CA Cancer J Clin 70:200–229. 10.3322/caac.21605 32275330 10.3322/caac.21605

[CR14] German Cancer Society (2022) German Guideline Program in Oncology (German Cancer Society, German Cancer Aid, AWMF): Soft Tissue Sarcoma Long version 1.1, 2022, AWMF Registration Number: 032/044OL

[CR15] Gronchi A, Miah AB, Dei Tos AP et al (2021) Soft tissue and visceral sarcomas: ESMO–EURACAN–GENTURIS clinical practice guidelines for diagnosis, treatment and follow-up. Ann Oncol 32:1348–1365. 10.1016/j.annonc.2021.07.006 34303806 10.1016/j.annonc.2021.07.006

[CR16] Hernández-Flores LA, López-Martínez JB, Rosales-de-la-Rosa JJ et al (2025) Assessment of challenging oncologic cases: a comparative analysis between ChatGPT, Gemini, and a multidisciplinary tumor board. J Surg Oncol. 10.1002/jso.28121 39936586 10.1002/jso.28121

[CR17] Horesh N, Emile SH, Gupta S et al (2025) Comparing the management recommendations of large language model and colorectal cancer multidisciplinary team: a pilot study. Dis Colon Rectum 68:41–47. 10.1097/DCR.0000000000003504 39679608 10.1097/DCR.0000000000003504

[CR18] Jakob J, Andreou D, Bedke J et al (2023) Ten recommendations for sarcoma surgery: consensus of the surgical societies based on the German S3 guideline “Adult Soft Tissue Sarcomas.” Langenbecks Arch Surg 408:272. 10.1007/s00423-023-03002-3 37430129 10.1007/s00423-023-03002-3 PMC10333354

[CR19] Jakob J, Hohenberger P (2016) Role of isolated limb perfusion with recombinant human tumor necrosis factor α and melphalan in locally advanced extremity soft tissue sarcoma. Cancer 122:2624–2632. 10.1002/cncr.29991 27197621 10.1002/cncr.29991

[CR20] Kalaiselvan R, Malik AK, Rao R et al (2019) Impact of centralization of services on outcomes in a rare tumour: retroperitoneal sarcomas. Eur J Surg Oncol 45:249–253. 10.1016/j.ejso.2018.06.032 30082178 10.1016/j.ejso.2018.06.032

[CR21] Karabuğa B, Karaçin C, Büyükkör M et al (2025) The role of artificial intelligence (ChatGPT-4o) in supporting tumor board decisions. JCM 14:3535. 10.3390/jcm14103535 40429531 10.3390/jcm14103535 PMC12112035

[CR22] Li C-P, Jakob J, Menge F et al (2024) Comparing ChatGPT-3.5 and ChatGPT-4’s alignments with the German evidence-based S3 guideline for adult soft tissue sarcoma. iScience. 10.1016/j.isci.2024.111493 39759026 10.1016/j.isci.2024.111493 PMC11699281

[CR23] Li C-P, Jia W-W, Chu Y et al (2025) Improving accuracy and source transparency in responses to soft tissue sarcoma queries using GPT-4o enhanced with German evidence-based guidelines. Oncol Res Treat. 10.1159/000544978 40024240 10.1159/000544978

[CR24] Meta AI (2024) Llama 3.2: Revolutionizing edge AI and vision with open, customizable models. https://ai.meta.com/blog/llama-3-2-connect-2024-vision-edge-mobile-devices/. Accessed 21 Mar 2025

[CR25] Napolitano A, Thway K, Huang P, Jones RL (2024) Centralisation of care improves overall survival for sarcoma patients. Ann Oncol 35:338–339. 10.1016/j.annonc.2024.01.009 38342185 10.1016/j.annonc.2024.01.009

[CR26] Peeperkorn M, Kouwenhoven T, Brown D, Jordanous A (2024) Is temperature the creativity parameter of large language models?

[CR27] Perez-Lopez R, Ghaffari Laleh N, Mahmood F, Kather JN (2024) A guide to artificial intelligence for cancer researchers. Nat Rev Cancer 24:427–441. 10.1038/s41568-024-00694-7 38755439 10.1038/s41568-024-00694-7

[CR28] Reis M, Reis F, Kunde W (2024) Influence of believed AI involvement on the perception of digital medical advice. Nat Med 30:3098–3100. 10.1038/s41591-024-03180-7 39054373 10.1038/s41591-024-03180-7 PMC11564086

[CR29] Roohani S, Handtke J, Hummedah K et al (2025) The sarcoma ring trial: a case-based analysis of inter-center agreement across 21 German-speaking sarcoma centers. J Cancer Res Clin Oncol 151:30. 10.1007/s00432-024-06063-z 39755880 10.1007/s00432-024-06063-z PMC11700044

[CR30] Strönisch A, Märdian S, Flörcken A (2023) Centralized and interdisciplinary therapy management in the treatment of sarcomas. Life 13:979. 10.3390/life13040979 37109507 10.3390/life13040979 PMC10144040

[CR31] Tirotta F, Bacon A, Collins S et al (2023) Primary retroperitoneal sarcoma: a comparison of survival outcomes in specialist and non-specialist sarcoma centres. Eur J Cancer 188:20–28. 10.1016/j.ejca.2023.04.004 37178646 10.1016/j.ejca.2023.04.004

[CR32] Valentini M, Szkandera J, Smolle MA et al (2024) Artificial intelligence large language model ChatGPT: is it a trustworthy and reliable source of information for sarcoma patients? Front Public Health 12:1303319. 10.3389/fpubh.2024.1303319 38584922 10.3389/fpubh.2024.1303319 PMC10995284

